# Supplementary material for: All-optical dissipative discrete time crystals
Source: Nat Commun. 2022 Feb 14;13:848. doi: 10.1038/s41467-022-28462-x (PMC8844012; doi:10.1038/s41467-022-28462-x)
Supplement: Supplementary file 1 — Supplementary Information [file 41467_2022_28462_MOESM1_ESM.pdf]

# Supplementary Information for "All-Optical Dissipative Discrete Time Crystals"

Hossein Taheri,[1] Andrey B. Matsko,[2] Lute Maleki,[3] and Krzysztof Sacha[4]

[1]*Department of Electrical and Computer Engineering, University of California Riverside, 3401 Watkins Drive, Riverside, CA 92521*

[2]*Jet Propulsion Laboratory, California Institute of Technology, 4800 Oak Grove Drive, Pasadena, California 91109-8099, USA*

[3]*OEwaves Inc., 465 North Halstead Street, Suite 140, Pasadena, CA, 91107, USA*

[4]*Instytut Fizyki Teoretycznej, Uniwersytet Jagielloński, ulica Profesora Stanisława Łojasiewicza 11, PL-30-348 Kraków, Poland*

This document includes Supplementary Information (SI) to accompany "All-Optical Dissipative Discrete Time Crystals." Translation symmetry properties of the equations of motion describing a Kerr cavity pumped by a single continuous-wave (CW) laser are reviewed in reference to discrete time crystals (DTCs). It is shown that in a monochromatically pumped resonator, dispersive wave (DW) emission or optical Cherenkov radiation emerging from high-order dispersion (HOD) or avoided mode crossings (AMCs) do not define discrete time translation symmetry (DTTS) in the equations governing microcomb dynamics, thus precluding DTC formation in these systems. We also include more details on the experimental implementation of the DTCs described in the main text, as well as an investigation into the role of dual pumping in the presence of disruptions appearing in the microresonator dispersion profile near the pumps. The latter analysis shows the dominant effect of the pumps overshadowing the detrimental impact of AMCs.

## I. ABSENCE OF DTTS IN MONOCHROMATICALLY-PUMPED MICROCOMBS

We expound in this section why monochromatically pumped microcombs do not constitute DTCs. This applies also to microcomb soliton trapping observed when certain group velocity dispersion (GVD) profiles (e.g., sign change in the residual dispersion or AMC) create high-power harmonics away from the pump in the comb spectrum. In a driven system, a DTC can arise only when DTTS exists and is broken by a periodic observable whose periodicity is an integer multiple $m > 1$ of that of the drive. In monochromatically pumped DW-emitting solitons and Kerr microcomb soliton crystals, the trapping potential originates from resonator properties (e.g., geometry and dispersion). By inspecting the Lugiato-Lefever equation (LLE) and its germane variants, we show that such effects do not establish any DTTS in the Kerr Hamiltonian, nor in the governing equations of motion. As a corollary, speaking of DTTS breaking in such systems is unjustified.

It should be stressed that when referencing time translation symmetry breaking (TTSB) in monochromatically-pumped microcombs, the observable to base symmetry breaking assessment on is scarcely identified in explicit terms. One can readily show that irrespective of the specific chosen observable, such systems cannot support DTCs. For instance, if photon count at a fixed position is being observed, *continuous* time translation symmetry exists at the input, because the quasi-classical (or coherent) state of the continuous wave (CW) pump laser has Poissonian photon statistics. But then, when a soliton is generated in the output, photon count probability will increase near the soliton peak. As a result, *continuous* TTSB occurs, which clearly does not incorporate DTC formation. If, on the other hand, the response observable is the electric field, then the pump is periodic but the response, strictly speaking, is not (because of the carrier-envelope offset phase) [1–3]. Therefore, the output-over-input periodicity ratio is generally not an integer. This shows that, despite apparent similarities, a soliton or soliton crystal driven by one CW pump does not constitute a DTC.

The rapidly oscillating optical carrier frequency does not enter the LLE [4–7]. It is straightforward to verify that this equation possesses continuous, but not discrete, time translation symmetry. In non-dimensional form and *before* moving to a rotating reference frame, the equation reads

$$\frac{\partial u}{\partial \bar{t}} = \left(-1 + \mathrm{i}\alpha + d_1 \frac{\partial}{\partial \theta} + \mathrm{i}\frac{d_2}{2}\frac{\partial^2}{\partial \theta^2} - \mathrm{i}|u|^2\right) u + F, \tag{1}$$

where the pump $F$ and spatiotemporal field envelope $u$ are both normalized to the hyperparametric sideband generation threshold and $\bar{t}$ is the slow time normalized to twice the cavity photon lifetime. The normalization follows Ref. [5] and we underscore the use of extra minus signs in normalizing pump-resonance detuning $\alpha$, free spectral range (FSR) parameter $d_1$, and the GVD coefficient $d_2$. As in the main text, $\theta$ is the azimuthal angle around the circular

resonator and related to the fast time $\tau$ through $\theta = 2\pi\tau/T_{\mathrm{R}}$, $T_{\mathrm{R}}$ denoting the roundtrip time. It is clear that linear translations with arbitrary increments $\Delta\bar{t}$ and $\Delta\tau$ (equivalently, $\Delta\theta$) of the slow and fast time variables, $\bar{t} \to \bar{t} + \Delta\bar{t}$ and $\tau \to \tau + \Delta\tau$ ($\theta \to \theta + \Delta\theta$), leave Eq. (1) unchanged. Consequently, this equation possesses continuous time translation symmetry and solutions which fulfill such symmetry are expected. However, owing to the nonlinear term in Eq. (1), symmetry-preserving solutions can be unstable and instead symmetry-broken steady states may emerge. Spontaneous breaking of the continuous time translation symmetry by soliton formation does not amount to DTC generation because there is no DTTS defined by a time-periodic drive which can be violated. It is worth noting that these symmetry properties are maintained in the LLE, which describes the process in a co-rotating reference frame [2, 5].

Without removing the carrier frequency $f_{\mathrm{P}}$, the excitation term $F$ changes to $F\exp(\mathrm{i}2\pi f_{\mathrm{P}}\bar{t})$, which defines a DTTS $\bar{t} \to \bar{t} + T_{\mathrm{P}}$, in which $T_{\mathrm{P}} = 1/f_{\mathrm{P}}$ is the pump period. However, as noted above, considering the carrier-envelope offset frequency, the electric field of a spontaneously generated soliton is not periodic. In other words, the soliton envelop is periodic with the cavity roundtrip time $T_{\mathrm{R}}$, but the ratio $T_{\mathrm{R}}/T_{\mathrm{P}}$ is not an integer, because in a monochromatically pumped microcomb soliton train the pulse repetition rate and the pump frequency are fundamentally independent [8]. Moreover, it can be shown that the repetition rate of the pulse train generated with a CW pump always experiences phase diffusion due to quantum noise [9, 10]. Considering the symmetry properties of the equations of motion clarifies why entrainment behavior recently predicted theoretically in certain microcombs [11] and observed experimentally in mode-locked lasers [12] does not amount to DTC formation.

We note that the photon count probability perspective described above has proven to be a valuable one. Recent experiments have explained the timing jitter of soliton microcombs from this viewpoint and shown that, remarkably, the soliton can in essence be considered a particle with the characteristic position-momentum quantum uncertainty between two conjugate variables [9]. Indeed signatures of the quantum nature of solitons have previously been studied extensively in the context of fiber solitons; see, e.g., [13, 14]. From this perspective, the soliton can be considered a massive particle in its ground mechanical quantum state. The large number of photons in this case and the collective effect of photon interactions in the thermodynamic limit notably match the requirements of DTC behavior in Kerr microcombs such that with two-pump excitation, this approach can create DTCs, as we have demonstrated in the main text.

## II. ABSENCE OF DTTS IN MICROCOMBS WITH DISPERSIVE WAVES RESULTING FROM HIGHER-ORDER DISPERSION

Resonator residual or integrated dispersion, $D_{\mathrm{int}} = \omega_\eta - (\omega_0 + D_1\eta)$, can predict DW frequencies in the microcomb spectrum. In this expression, $\eta = j - j_0$ is the centered mode number, $\omega_0$ and $\omega_\eta$ signify the frequencies of the pumped mode ($j_0$) and a generic resonant mode corresponding to mode number $j$, $D_1/2\pi$ is the FSR at the pump frequency, and $D_2 = \omega_{j_0+1} + \omega_{j_0-1} - 2\omega_{j_0}$ denotes the GVD coefficient. The detuning of resonator modes from the grid defined by microcomb harmonics is minimized in the vicinity of residual dispersion sign change ($D_{\mathrm{int}} = 0$), creating high-power frequency harmonics (DWs) in the spectrum as a result of the optical analogue of Cherenkov radiation [15]. For narrow-band frequency combs and negligibly small third- or higher-order dispersion (HOD), DW emission cannot occur because $D_{\mathrm{int}} = D_2\eta^2/2$ does not cross zero away from the pump. However, in the presence of HOD the residual dispersion, $D_{\mathrm{int}} = D_2\eta^2/2 + D_3\eta^3/6 + \ldots$, can vanish for $\eta \neq 0$ to give rise to DWs [16]. The LLE in this case includes higher-order $\theta$-derivatives and takes the following form [17]

$$\frac{\partial u}{\partial \bar{t}} = \left(-1 + \mathrm{i}\alpha - \sum_{n=2}^{N>2} \mathrm{i}^{n+1}\frac{d_n}{n!}\frac{\partial^n}{\partial\theta^n} - \mathrm{i}|u|^2\right)u + F. \tag{2}$$

Again, Eq. (2) is invariant under linear translations with arbitrary increments $\Delta\bar{t}$ and $\Delta\tau$ of the slow and fast time variables, $\bar{t} \to \bar{t} + \Delta\bar{t}$ and $\tau \to \tau + \Delta\tau$. This equation is in the co-moving reference frame, and as noted before, the difference is merely the removal of the $d_1\partial u/\partial\theta$ term such that relevant symmetry properties remain unchanged. Therefore, Eq. (2) possesses continuous time translation symmetry; there is no DTTS and hence DTC formation cannot occur.

## III. ABSENCE OF DTTS IN MICROCOMB SOLITON CRYSTALS STABILIZED BY AVOIDED MODE CROSSINGS

The interaction between two mode families with different transverse profiles in a generic resonator is described by two coupled LLEs [18, 19]. The dynamics of microcomb formation when one of the two interacting mode families 1

and 2 is directly pumped (i.e., in a monochromatically-pumped resonator) is captured by

$$\frac{\partial u}{\partial \bar{t}} = \left( -\frac{\bar{\kappa}}{\kappa_2} + \mathrm{i}\alpha_1 + \delta_1^{(1)} \frac{\partial}{\partial \theta} + \mathrm{i}\frac{d_2^{(1)}}{2}\frac{\partial^2}{\partial \theta^2} - \mathrm{i}D^{(1,1)}|u|^2 - \mathrm{i}D^{(1,2)}|v|^2 \right) u + F_1, \tag{3}$$

$$\frac{\partial v}{\partial \bar{t}} = \left( -\frac{\bar{\kappa}}{\kappa_1} + \mathrm{i}\alpha_2 + \delta_1^{(2)} \frac{\partial}{\partial \theta} + \mathrm{i}\frac{d_2^{(2)}}{2}\frac{\partial^2}{\partial \theta^2} - \mathrm{i}D^{(2,1)}|u|^2 - \mathrm{i}D^{(2,2)}|v|^2 \right) v + F_2, \tag{4}$$

in which $u(\bar{t}, \theta)$ and $v(\bar{t}, \theta)$ are the spatiotemporal field envelopes, respectively, of mode 1 and 2, $\kappa_{1,2}$ is the resonance half width at half maximum (HWHM), $\bar{\kappa} = (\kappa_1 + \kappa_2)/2$ is the average HWHM, $\alpha_{1,2}$ is the detuning, $\delta_1^{(1,2)}$ is the group velocity mismatch of the envelope field with respect to the average group velocity $d_1^{(\mathrm{av})} = (d_1^{(1)} + d_1^{(1)})/2$, $d_1^{(1,2)}$ is the FSR for the respective mode family, $d_2^{(1,2)}$ is the GVD parameter, and $F_{1,2}$ is the effective pump field projected on each of the mode families. For a monochromatic pump tuned primarily to excite one mode family, one of the two drive terms $F_1$ or $F_2$ is often negligible; see, e.g., [20]. Coupling between the equations originates from the overlap integrals of the two modes $D^{(j,k)}$, $j, k \in \{1, 2\}$. The notation follows [18]. Like before, $\bar{t}$ is the slow time and the azimuthal angle $\theta$ is linearly proportional to the fast time $\tau$.

The set of coupled LLEs in Eq. (3) is invariant under linear translations with arbitrary increments $\Delta\bar{t}$ and $\Delta\tau$ of the slow and fast time variables, i.e., $\bar{t} \to \bar{t} + \Delta\bar{t}$ and $\tau \to \tau + \Delta\tau$. Therefore these equations possess continuous time-translation symmetry. In other words, the AMC-induced potential trapping solitons around the resonator does not define DTTS in the equations of motion and so the system cannot give rise to DTCs.

We conclude the discussion on the absence of DTTS in monochromatically-pumped microcombs by highlighting two distinctions offered by second pump exciting subharmonics between the two pumps. Temperature change will shift the modes of a cavity and hence the frequency of the DW or AMC signature in the microcomb power spectrum [21]. As a result, a laser pumping another nearby mode at a fixed frequency or even one pumping the same temperature-shifted mode will in general create a periodic potential with a different periodicity around the cavity. This periodicity is not tied to the pump frequency, and as shown above, does not change the continuous temporal symmetry of the governing equations, nor does it establish any DTTS. In the two-pump system, in contrast, the excitation frequency and the DTTS it defines are always set by the drive, i.e., the beating of the two pumps, so that even with temperature-shifted modes, the same driving frequency can readily be realized. This driving frequency dictates the DTTS of the system, as evidenced by the two panels in Fig. 2 (main text). Additionally, in a singly-pumped microresonator setup with a fixed laser frequency, a microcomb soliton whose span does not cover the spectral position of the more energetic DW harmonic can simply be generated by reducing the pump power and consequently the comb span. In this case, the CW background will not be modulated at all. So, in the same resonator and with the same pump frequency, no periodic potential will form around the resonator, and no soliton trapping and crystallization will occur [22]. In contrast, in the dichromatically-pumped system, DTTS is still present even if the pumps are weak and excite no subharmonics. This is a fundamental distinction, and an essential one, in defining DTCs. It is noteworthy that subharmonic generation in a dual-pump Kerr cavity is possible both in the normal and anomalous dispersion regimes [23–25].

## IV. ABSENCE OF STRONG DISPERSIVE WAVE PINNING IN THE EXPERIMENTS

An AMC arises from coupling between different cavity spatial mode families and its signature in the frequency comb spectrum consists of an energetic harmonic. In our experiments, instead of DW-like pinning at a fixed frequency, high-power comb harmonics follow the external pump frequencies in subsequent 2-, 3- and 4-FSR pump separations; see below. Consequently, unlike microcomb soliton crystals whose stability depends on AMCs, subharmonic generation data presented in the main text are not affected by AMCs.

To elaborate, an AMC pins a high-power harmonic in the microcomb spectrum at the position of the mode crossing [26]. While AMCs are practically inevitable in experiments, whether or not they impact the excitation and stability of a microcomb generated in a certain region can partially be judged based on the existence of strong disruptions in the comb teeth pinned in the spectrum and fixed as the pump frequency is shifted. This pinning signature is negligibly weak in our experiments, both when the two pumps generate harmonics through four-wave mixing (FWM), Supplementary Figure 1, and when they excite subharmonics and the beatnote of the pumps is changed in successive FSRs in the same pumping region, Supplementary Figure 2. This is particularly manifest in the spectrum for 2-FSR separation of the pumps, Supplementary Figure 2(e), which shows no notable sign of an AMC. Instead, high-power harmonics always follow the external pumps and the FWM harmonics that appear at frequency intervals dictated by their beatnote. Therefore, subharmonic generation in our experiments is not impacted by AMC and mode crossings play no role in stabilizing the observed microcombs. This fact is supported also by our numerical modeling data which demonstrates stable pulse train propagation over hundreds of cavity photon lifetimes (thousands of drive periods); see

Figs. 3(a, d) in the main text. We have not incorporated any AMC or GVD parameter beyond $D_2$ in our numerical simulations.

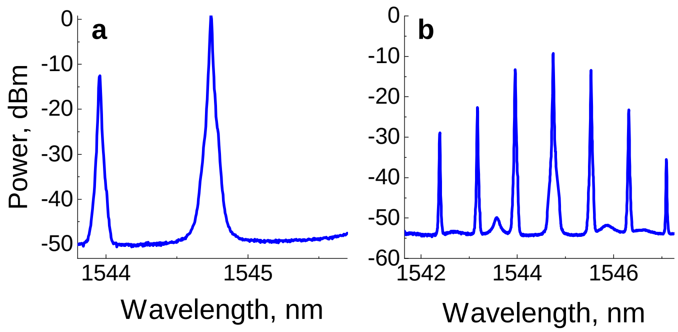


**Supplementary Figure 1.** Experimental data illustrating the two pumps (a) and four-wave mixing creating harmonics spaced by their beatnote (b). Compared to Supplementary Figure 2, pump parameters are chosen such that subharmonic generation does not occur. No evidence of AMC-induced frequency pinning is observed.

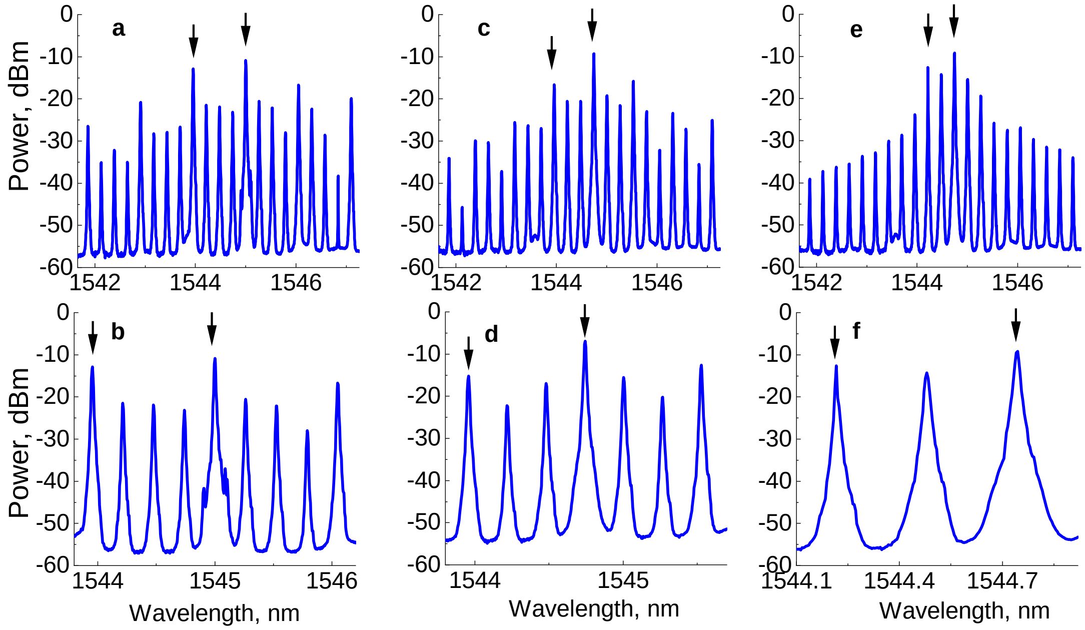


**Supplementary Figure 2.** Experimental data demonstrating stable subharmonic generation for different successively decreased pump beatnotes, (a, b) 4, (c, d) 3, and (e, f) 2 FSRs, in the same pumping region. Each bottom panel is a zoomed-in version of the panel above it. No significant AMC signature or harmonic pinning is observed and high-power microcomb harmonics follow the external pumps. Experimental parameters are the same as those described in the main text (see Methods).

## V. SUPPRESSION OF MODE DISRUPTIONS BY THE TWO PUMPS AND THE DESTABILIZING IMPACT OF MODE ANTI-CROSSINGS

We had noted in the main text that our experiments were performed in a regime where none of the pumps could generate solitons independently (see Methods). This is verified in the comb energy vs. detuning curve of Supplementary Figure 3(a). This figure illustrates that with a single pump, the soliton formation regime is limited to a narrow sliver (the step-like region) and that in experiments we operated to the right of this region, indicated by the red arrow and dotted vertical line, where the pump cannot generate any solitons. Supplementary Figure 3(b), further discussed below, depicts the same plot as in Supplementary Figure 3(a) but with the addition of an AMC near the

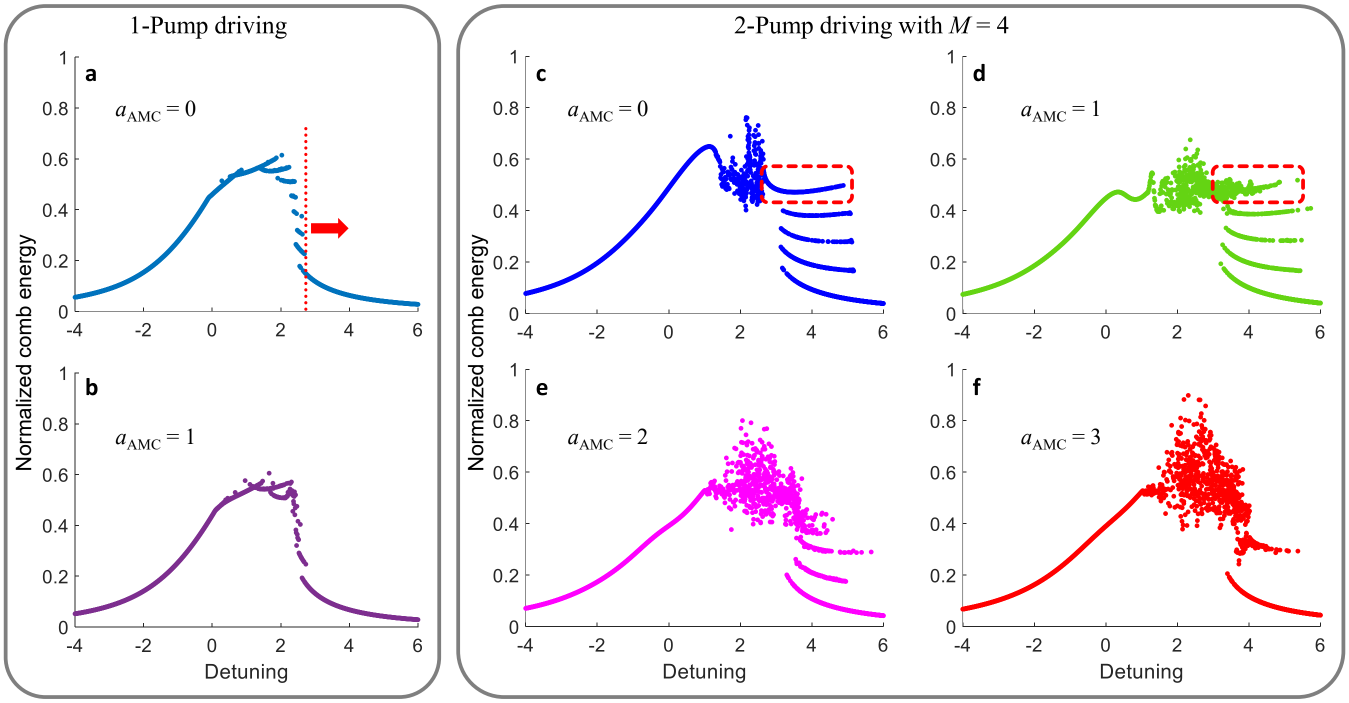


**Supplementary Figure 3.** Investigation of the impact of AMC on subharmonic generation for 4 FSRs between the two pumps. Each curve shows the total intra-cavity comb energy vs. detuning at steady state (i.e., each data point on each curve refers to one run till steady state, if existent, was reached). (a, b): Single-pump driving without an AMC (a) and with an AMC parametrized with $a_{\rm AMC} = 1$ (normalized) and $b = -4$ (b); see text for details. As noted in the main text (see Methods), the operation region of our system was to the right of the vertical dotted line in (a), where a single pump cannot sustain stable soliton formation. (c-f): Double-pump driving without (c) and with (d-f) the AMC at $b = -4$. In (d-f) the strength of the AMC is increased from $a_{\rm AMC} = 1$ in (d), to 2 in (e), and 3 in (f). As the AMC grows stronger, it leads to the destabilization of the solitons, evident here as the replacement of clean soliton steps by wider regions where the chaotic power of the unstable microcomb may land after hundreds of cavity lifetimes. An example is highlighted by the boxed regions (dashed red) in (c) and (d), where the top-most step has vanished in (d). In (e) and (f) one and two other high-energy steps have vanished as well.

pumps. System parameters in Supplementary Figures 3(a,b) match those of our experiments, with the pump power corresponding to the stronger of the two pumps in the case of monochromatic driving. The weaker pump, as noted in the text, is slightly sub-threshold and hence does not obviously support hyperparametric sideband generation or soliton formation. It should be reiterated that, as shown in Sections I and III above, a monochromatically-pumped Kerr cavity, with or without AMC, does not possess discrete time-translation symmetry and hence cannot accommodate DTCs.

Supplementary Figure 3(c) shows how vastly different the behavior of the system becomes with the addition of the second pump. In this example, the second pump is added 4 FSRs away from the first one ($M = 4$). Instead of 8 very narrow steps in Supplementary Figure 3(a), $M + 1 = 5$ wide steps are clearly visible in the figures, as we expected from the theory described in the text. The lowermost step corresponds to no solitons, and the upper ones to 1, 2, 3, and 4 solitons per round-trip time, respectively. All steps are very well-defined with successive runs of the numerical simulation with random initial conditions all resulting in the same steady states with the same comb power and intra-cavity energy at each realization.

In all other panels in Supplementary Figure 3, i.e., panels (b, d, e, f), an AMC is added in the numerical simulations. We have used the model used in Ref. [27] to implement the mode crossing. In this model, the parameter $a$ indicates a measure of the "strength" of the mode crossing and we have represented it with $a_{\rm AMC}$ (normalized to the cavity FWHM) in Supplementary Figure 3, while parameter $b$ (non-dimensional) determines its mode number (essentially, frequency). In panels (b, d-f) of Supplementary Figure 3, $b = -4$. (Note that all of our experimental data are reported vs. wavelength, not frequency.) Without a second pump and with $a_{\rm AMC} = 1$, Supplementary Figure 3(b), some of the steps in the narrow soliton formation region of Supplementary Figure 3(a) show clear signs of destabilization. This observation matches findings previously reported in the literature, particularly that AMCs very close to the pump destabilize microcombs and hinder soliton formation; see, e.g., [27].

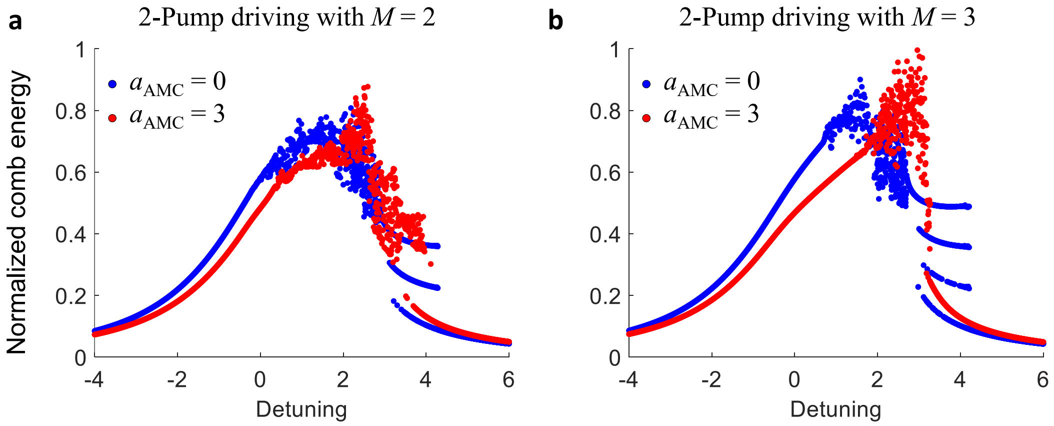


**Supplementary Figure 4.** Investigation of the impact of AMC on subharmonic generation, similar to Supplementary Figure 3, but for 2 (a) and 3 (b) FSRs between the two pumps.

In Supplementary Figures 3(d, e, f), $a_{\mathrm{AMC}}$ is increasing from 1 to 3, respectively. With two-pump driving and the small value of $a_{\mathrm{AMC}} = 1$, even though the lower-energy steps do not change much, destabilization of the solitons starts to occur noticeably. The destruction of stable soliton steps is especially clear from the top-most step (boxed with a red dashed-line rectangle) and would translate into large phase noise for the subharmonic beatnote. Supplementary Figures 3(e, f), where $a_{\mathrm{AMC}} = 2$ and 3, demonstrate how by increasing the strength of the AMC, soliton steps are almost completely washed away, leaving only the bottom step (no solitons) and a fuzzy trace of the destabilized third step in panel (f). The trend is clear: further increase of the AMC strength would completely eradicate soliton steps. It is worth noting that with smaller $a_{\mathrm{AMC}}$ (e.g., $a_{\mathrm{AMC}} = 0.5$) the differences with the AMC-free case were unremarkable, indicating that the AMC is completely subjugated by the cooperation of the two pumps, as if the mode disruptions did not exist at all. It should be emphasized that the same destabilizing effect of AMCs is observed (and is even more severe) for the same cavity modal profile and AMC frequency and with $M = 2$ and 3, as the overlaid plots in Supplementary Figure 4 show. (Not included herein, we have observed the same disruptive influence of AMCs at other values of $b$ as well, e.g., for the AMC 5 FSRs away from the stronger pump, $b = -5$.)

The results discussed above show that AMCs, especially those so close to the pumps, would only hurt, and not assist, subharmonic generation. In other words, the existence of AMCs would hinder the emergence of the stable and low phase noise subharmonics that herald DTC formation. In our experiments, we have observed stable microcombs with robust subharmonics in all of the discussed cases ($M = 2, 3$, and 4), which would not have been possible if the AMCs were dominant. This observation confirms that AMCs in our experiments were indeed weak and completely overshadowed by the pumps.

## SUPPLEMENTARY REFERENCES


[1] S. T. Cundiff, Phase stabilization of ultrashort optical pulses, Journal of Physics D: Applied Physics **35**, R43 (2002).

[2] A. Kovach, D. Chen, J. He, H. Choi, A. H. Dogan, M. Ghasemkhani, H. Taheri, and A. M. Armani, Emerging material systems for integrated optical Kerr frequency combs, Adv. Opt. Photon. **12**, 135 (2020).

[3] H. Taheri and A. B. Matsko, Dually-pumped Kerr microcombs for spectrally pure radio frequency signal generation and time-keeping, in *Laser Resonators, Microresonators, and Beam Control XXI*, Vol. 10904 (International Society for Optics and Photonics, 2019) p. 109040P.

[4] Y. Chembo and N. Yu, Modal expansion approach to optical-frequency-comb generation with monolithic whispering-gallery-mode resonators, Phys. Rev. A **82**, 033801 (2010).

[5] Y. Chembo and C. Menyuk, Spatiotemporal Lugiato-Lefever formalism for Kerr-comb generation in whispering-gallery-mode resonators, Phys. Rev. A **87**, 053852 (2013).

[6] A. Matsko, A. Savchenkov, W. Liang, V. Ilchenko, D. Seidel, and L. Maleki, Mode-locked Kerr frequency combs, Opt. Lett. **36**, 2845 (2011).

[7] S. Coen, H. Randle, T. Sylvestre, and M. Erkintalo, Modeling of octave-spanning Kerr frequency combs using a generalized mean-field Lugiato-Lefever model, Opt. Lett. **38**, 37 (2013).

[8] A. B. Matsko and L. Maleki, On timing jitter of mode locked Kerr frequency combs, Optics Express **21**, 28862 (2013).

[9] C. Bao, M.-G. Suh, B. Shen, K. Şafak, A. Dai, H. Wang, L. Wu, Z. Yuan, Q.-F. Yang, A. B. Matsko, F. K. Kärtner, and K. J. Vahala, Quantum diffusion of microcavity solitons, Nature Physics **17**, 462 (2021).

[10] A. B. Matsko and L. Maleki, Noise conversion in Kerr comb RF photonic oscillators, JOSA B **32**, 232 (2015).

[11] D. C. Cole and S. B. Papp, Subharmonic Entrainment of Kerr Breather Solitons, Physical Review Letters **123**, 173904 (2019).

[12] T. Xian, L. Zhan, W. Wang, and W. Zhang, Subharmonic Entrainment Breather Solitons in Ultrafast Lasers, Physical Review Letters **125**, 163901 (2020).

[13] L. A. Lugiato and F. Castelli, Quantum noise reduction in a spatial dissipative structure, Physical Review Letters **68**, 10.1103/PhysRevLett.68.3284 (1992).

[14] P. D. Drummond, R. M. Shelby, S. R. Friberg, and Y. Yamamoto, Quantum solitons in optical fibres, Nature **365**, 307 (1993).

[15] V. Brasch, M. Geiselmann, T. Herr, G. Lihachev, M. H. P. Pfeiffer, M. L. Gorodetsky, and T. J. Kippenberg, Photonic chip–based optical frequency comb using soliton Cherenkov radiation, Science **351**, 357 (2016).

[16] C. Bao, H. Taheri, L. Zhang, A. Matsko, Y. Yan, P. Liao, L. Maleki, and A. Willner, High-order dispersion in Kerr comb oscillators, JOSA B **34**, 715 (2017).

[17] M. Lamont, Y. Okawachi, and A. Gaeta, Route to stabilized ultrabroadband microresonator-based frequency combs, Opt. Lett. **38**, 3478 (2013).

[18] G. D'Aguanno and C. R. Menyuk, Nonlinear mode coupling in whispering-gallery-mode resonators, Physical Review A **93**, 043820 (2016).

[19] M. Karpov, M. H. P. Pfeiffer, H. Guo, W. Weng, J. Liu, and T. J. Kippenberg, Dynamics of soliton crystals in optical microresonators, Nature Physics **15**, 1071 (2019).

[20] A. Matsko, W. Liang, A. Savchenkov, D. Eliyahu, and L. Maleki, Optical Cherenkov radiation in overmoded microresonators, Optics Letters **41**, 2907 (2016).

[21] Y. Wang, F. Leo, J. Fatome, M. Erkintalo, S. G. Murdoch, and S. Coen, Universal mechanism for the binding of temporal cavity solitons, Optica **4**, 855 (2017).

[22] H. Taheri, *Ultrashort pulses in optical microresonators with Kerr nonlinearity*, PhD Thesis, Georgia Institute of Technology (2017).

[23] T. Hansson and S. Wabnitz, Bichromatically pumped microresonator frequency combs, Phys. Rev. A **90**, 013811 (2014).

[24] Y. Okawachi, M. Yu, K. Luke, D. O. Carvalho, S. Ramelow, A. Farsi, M. Lipson, and A. L. Gaeta, Dual-pumped degenerate Kerr oscillator in a silicon nitride microresonator, Optics Letters **40**, 5267 (2015).

[25] H. Taheri and A. Matsko, Crystallizing Kerr Cavity Pulse Peaks in a Timing Lattice, in *Frontiers in Optics + Laser Science APS/DLS (2019), paper JTu3A.90* (Optical Society of America, 2019).

[26] X. Xue, M. Qi, and A. M. Weiner, Normal-dispersion microresonator Kerr frequency combs, Nanophotonics **5**, 244 (2016).

[27] T. Herr, V. Brasch, J. D. Jost, I. Mirgorodskiy, G. Lihachev, M. L. Gorodetsky, and T. J. Kippenberg, Mode spectrum and temporal soliton formation in optical microresonators, Phys. Rev. Lett. **113**, 123901 (2014).
